# Adolescents’ time-use and academic attainment: A longitudinal, compositional analysis in the Millennium Cohort Study

**DOI:** 10.1371/journal.pone.0346302

**Published:** 2026-04-09

**Authors:** Ciaran M. C. Maloney, Andrew J. Atkin, Lee C. Beaumont, Jack R. Dainty, Victoria E. Warburton

**Affiliations:** 1 School of Education and Lifelong Learning, University of East Anglia, Norwich, United Kingdom; 2 School of Health Sciences, University of East Anglia, Norwich, United Kingdom; 3 Norwich Medical School, University of East Anglia, Norwich, United Kingdom; Swansea University, UNITED KINGDOM OF GREAT BRITAIN AND NORTHERN IRELAND

## Abstract

There is a lack of longitudinal evidence regarding the associations between 24-hour movement behaviours and academic attainment. Using the Millennium Cohort Study, we examined the association of time-use diary assessed movement behaviours at age 14 years with formal school examination results at age 16. Analytical samples for weekday and weekend analyses were n = 1644 and n = 1642, respectively (weekday sample at baseline: 54% female, 80% White British). Time-use diary data were used to derive six behavioural sets: (a) sleep; (b) physical activity; (c) electronic media; (d) school-related activities; (e) hobbies and socialising; and (f) domestic tasks, personal care, and work-related activities. The primary outcome was Attainment 8, a composite marker of overall academic attainment across a range of subjects devised by the Department for Education in England. Multivariable compositional isotemporal substitution models were used to estimate differences in Attainment 8 associated with reallocating time between behaviour sets. Predicted differences in Attainment 8 scores were statistically significant for all models (weekday and weekend) that simulated the addition or removal of sleep, with an increase in sleep duration associated with lower academic attainment. Reallocating 20 minutes to sleep from physical activity was associated with the largest reduction in Attainment 8 (β = −.81, 95%CI [−.87, −.76]), while comparable reallocations to physical activity from sleep were associated with enhanced Attainment 8 (β = .29, 95%CI [.24,.34]). Associations were largely consistent between week and weekend days but remained small in magnitude across all behaviour sets. Reallocations to sleep from any other behavioural set was adversely associated with academic attainment, while comparable increases in physical activity or hobbies were beneficially associated with academic attainment. Time-use during mid-adolescence may be associated with later academic attainment, but associations are behaviour-specific and small in magnitude, warranting further exploration prior to intervention development.

## Introduction

Academic attainment, defined as the highest level of education completed [1], is a multifaceted construct shaped by a myriad of personal, environmental, and institutional factors [2]. High academic attainment is associated with enhanced employment [3] and health-related outcomes [4] in later life. However, recent evidence suggests that General Certificate of Secondary Education (GCSE) scores, the standard academic qualification for students aged 16 in the United Kingdom (UK), have decreased for core subjects such as mathematics and English since 2018 [5]. Middle adolescence (aged 14–17 [6]) represents a key educational transition period, characterised by increasing academic demands and formal assessments through GCSEs. This period of rapid physical, mental, and social development is also when behavioural habits are established that may persist into adulthood [7]. Against this backdrop, researchers and educators are increasingly focused on identifying and implementing effective strategies to enhance academic attainment.

A growing body of evidence indicates that individual movement behaviours, such as physical activity, sleep, and sedentary behaviour, may be associated with academic attainment [8–10]. For example, female students who engaged in higher levels of moderate-to-vigorous physical activity at age 11 achieved higher grades in science assessments at ages 11 and 16 years [11]. Moreover, a population-based cross-sectional study of 7798 Norwegian adolescents (aged 16–19 years) found that 7–9 hours of sleep per night was associated with higher grade point averages [12]. In recent years, however, the focus of behavioural epidemiology research has shifted away from studying individual behaviours in isolation to an integrative approach, recognising that behaviours are co-dependent within a 24-hour period [13]. This transition is reflected in public health guidelines that recommend young people (aged 5–17 years) accumulate at least 60 minutes of physical activity per day, seven hours of sleep per night, and limit recreational screen time to two hours [14].

Existing cross-sectional studies have suggested that adherence to movement behaviour guidelines is associated with enhanced academic attainment [15–18]. In a sample of approximately 2000 Australian children aged 11–12 years, for example, self-reported adherence to the physical activity, sleep, and screen time guidelines was associated with better literacy performance compared to children who did not meet any guidelines [19]. However, studies that focus on meeting movement behaviour guidelines, which reduce the behavioural exposures to binary variables, are limited as they do not inform children, parents, or practitioners how much time they should spend on different behaviours or capture the nuance of how different behaviours interact [20]. Increased recognition of the interplay between behaviours as a potential determinant of health or other important outcomes has driven the uptake of analytical approaches, such as compositional isotemporal substitution modelling, that can be used to examine how reallocating time between different behaviours may impact health or academic outcomes, while retaining exposure variables in their continuous form [21,22].

A recent scoping review [23] indicated that much of the existing work using compositional isotemporal substitution modelling in young people has relied on cross-sectional data and focused on health-related outcomes, such as mental health [24] and adiposity [25]. However, given that academic attainment is associated with later-life benefits such as better employment prospects [3] and health-related outcomes [4], examining how reallocations of movement behaviours are associated with academic attainment may provide insights into adolescent development. A recent cross-sectional study reported that reallocating 60 minutes from screen time to physical activity was associated with a 0.9% increase in National Assessment Program for Numeracy and Literacy (NAPLAN) writing scores for children aged 11–12 years [26]. Additionally, a study of 43,616 ‘sleep-restricted’ (<8 hours of sleep per night) secondary school-aged Chinese students found that reallocating 50 minutes of self-reported gaming to sleep was associated with improved academic performance [27]. Building upon this foundation of cross-sectional research, longitudinal studies are valuable to establish the timeframe in a young person’s life when modifications to their movement behaviour may impact their academic attainment. Therefore, the aim of the present study was to examine the association of movement behaviours assessed at age 14 years with formal school examination results achieved at age 16.

## Materials and methods

### Sample and data collection

Data were from the Millennium Cohort Study (MCS), an observational cohort study of the physical, emotional, economic, cognitive, behavioural, and health-related circumstances of children born in the UK between September 2000 and January 2002 [28]. In total, 18,522 families (18,818 children) were recruited in the first assessment; a further eight sweeps of assessment have been completed (age 9 months, 3, 5, 7, 11, 14, 17, and 23 years). This study uses data from sweeps six (MCS6; data collection: January 2015-April 2016 [29]) and seven (MCS7; data collection: January 2018-March 2019 [30]), when participants were 14- and 16-years-old, respectively. Ethical approval for the sixth sweep (MCS6) was provided by the National Research Ethics Service (NRES) Research Ethics Committee (REC) London (Central ref: 13/LO/1786). Ethical approval for MCS7 was obtained from the NRES REC Northeast – York (ref: 17/NE/0341). Anonymised data was accessed free of charge from the UK Data Service (http://doi.org/10.5255/UKDA-SN-8156-7) on 1^st^ October 2023.

### Assessment of time-use

At MCS6, participants were invited to complete a time-use diary for two days (one weekday and one weekend day), randomly selected by computer assisted software. From 4642 participants, 69% (n = 3202) used a phone application diary format, 25% (n = 1157) used the online version, and 6% (n = 283) used a paper diary. Participants recorded their behaviour in 10-minute timeslots from 4am to 4am the following day. The 4am-4am measurement period is a commonly applied timeframe for collection of time-use data, achieving an optimal balance between time coverage and respondent burden [31]. In each timeslot, participants indicated their main activity from a pre-specified list of 44 activities nested within 12 categories. Categories included ‘sleep and personal care’, ‘school, homework, and education’, ‘physical exercise and sports’, and ‘internet, television, and digital media’. Based on previous research [24], weekday activities were recoded into six mutually exclusive activity sets (S1 Table): (a) sleep; (b) physical activity; (c) electronic media; (d) school-related activities; (e) hobbies and socialising; (f) domestic tasks, personal care, and work-related activities. For the weekend data, four sets were used (sleep; electronic media; hobbies and socialising; and domestic tasks, personal care, and work-related activities) because of the limited time spent in school and physical activities. These activities accounted for the entirety of participants’ daily time-use (24hour/1440 minutes). The sleep component represented all sleep occurring between 4 am and 4 am; as such it may not embody a full overnight sleep and includes naps taken during the day.

Diaries (days) with missing data (one or more timeslots with no activity indicated) were excluded from the analysis, as were diaries with no entries for ‘sleep’ or ‘domestic’ activities (which included getting dressed and eating), as these were deemed unreliable accounts of a complete day’s behaviour [24]. The presence of zeros hampers the application of compositional data analysis as log-ratios that include a zero value cannot be computed [32]. Zero values in any activity component were replaced with small positive values (<10 minutes), with time proportionally reallocated from the remaining non-zero components to preserve a total time of 1440 minutes, consistent with previous work [33]. This approach, known as multiplicative replacement, preserves the compositional structure of the data and enables the use of isometric log ratio (ILR) transformations in subsequent statistical analyses.

### Academic attainment

Information on participants’ academic attainment was obtained from The National Pupil Database (NPD [34]). The NPD is an administrative data resource curated by the UK Government for funding purposes, school performance tables, policymaking, and research; it contains school-level data on all pupils in state schools in England [35]. GCSE grades and other school-level data were available from 8204 pupils. GCSEs are qualifications obtained in three of the four UK countries (England, Wales, and Northern Ireland) between the ages of 14–16 years, with examination typically sat at the end of Year 11 in secondary school [36]. In the MCS, NPD data was only available for participants residing in England. Academic attainment was measured using Attainment 8, a composite marker of overall academic attainment across a range of subjects devised by the Department for Education [37]. Attainment 8 is calculated by summing a pupil’s highest scores across eight government-approved subjects, with scores ranging from 0–90 [38]. Details of how Attainment 8 is calculated is available at: https://www.goodschoolsguide.co.uk/curricula-and-exams/progress-8-attainment-8.

### Covariates

Information on participants biological sex (male or female), ethnicity (self-reported; six category census classes: White British, Mixed, Indian, Pakistani or Bangladeshi, Black or Black British, and other ethnic groups), age (years), and biological maternal education (mother-reported; categorised into six levels: None, Overseas Qualification Only, NVQ1 [GCSE D-G], NVQ2 [GCSE A*-C], NVQ3 [A-level], NVQ4/5 [Degree]) was ascertained from MCS6. Participant’s eligibility for free school meals (school-reported, yes/no) was obtained from MCS7.

### Statistical analysis

Analyses were conducted using R open-source software. The packages zCompositions and robCompositions were used for the analysis of compositional data (R Version 2.0–8 [39]). Demographic characteristics of the analytical sample are presented as frequencies and percentages or means with standard deviations (SD), as appropriate. Differences in characteristics between participants included and excluded from the sample were compared using t-tests or X² tests. Raw time-use data containing zero values were summarised for each behavioural set using arithmetic mean and standard deviation. For the imputed time-use compositions, in which zeros were replaced with small positive values, time in each behavioural set is presented as the compositional mean, calculated as the geometric mean of each behaviour and linearly adjusted to sum to 1440 minutes. Summary statistics are presented separately for weekday and weekend data. Time-use compositions were expressed as sets of ILR coordinates. Once compositional data are transformed into ILR coordinates, they become compatible with operations in real space, enabling their inclusion in conventional statistical models, such as linear regressions [22]. The ILR transformation preserves the relative differences between components and produces orthogonal (non-multicollinear) coordinates [40]. Each ILR coordinate represents one behaviour relative to the remaining behaviours. For example, a composition with three parts is transformed into two ILR coordinates, ensuring that any increase in one behaviour is reflected by a proportional decrease in the remaining behaviours, consistent with the fixed 24-hour day.

Due to low geometric means of physical activity and school-related activities in the weekend analysis, meaningful reallocations of time could not be accommodated, and these behaviour sets were subsequently removed from the weekend analysis. The six-part (weekday) and four-part (weekend) compositions are expressed via five and three sets of ILR coordinates, respectively. We conducted compositional isotemporal substitution analyses to model the association between time-use behaviours and Attainment 8 scores. In this context, compositional isotemporal substitution analysis is used to estimate the effects on academic attainment of replacing a fixed amount of time in one behaviour set with the same amount of time in another behaviour set. We modelled fixed time reallocations of 20 minutes on weekdays and 30 minutes on weekend days between pairs of behaviour sets. All models were adjusted for sex, ethnicity, free school meals, and maternal education. Preliminary analyses showed no association between age and Attainment 8; thus, it was removed from the main analysis.

We explored the shape of the association between behaviour reallocations of differing durations and academic attainment outcomes. We modelled predicted differences in Attainment 8 scores for the time reallocations of −20 minutes to +20 minutes between all behaviour sets in 5-minute increments. Twenty minutes was selected as the duration of simulated reallocations, consistent with previous research, demonstrating behavioural exchanges of this duration are associated with academic outcomes [41]. As the data supported greater flexibility in time reallocations in the weekend data, we modelled predicted differences in Attainment 8 scores for reallocations of −30 minutes to +30 minutes in 5-minute increments. To assess the robustness of our analysis to different attainment metrics, we additionally conducted all analysis using ‘number of passes’ at GCSE (grades A*-C) as the outcome (S2 Table and S3 Table).

## Results

Data were available from 4626 diaries, obtained from 2489 participants. A total of 308 participants with missing covariate data were removed. Due to missing time-use diary data or non-reporting of sleep or domestic/personal activities, a further 367 and 398 participants were removed from the weekday and weekend analyses, respectively. The analytical samples for weekday and weekend analyses were n = 1644 and n = 1642 participants. In total, 1875 participants were included in the analyses with 1411 participants providing both weekday and weekend data. A flow chart of participant inclusion is available in S1 Fig.

### Participant characteristics

Characteristics of participants included in the weekday and weekend analyses are presented in Table 1. Overall, the weekday sample was 13.8 years old (SD = .5) at baseline, 54% female, and predominantly White British (80%). Compared to those excluded from the analysis, participants in the weekday and weekend samples were more likely to be of white ethnicity (weekday: 80%, weekend: 80%, excluded: 72%; p < .001) and less likely to claim free school meals (weekday: 7%, weekend: 7%, excluded: 15%; p < .001), but there were no differences in age, sex, or maternal education.

**Table 1:** Participant Characteristics. Values are n (%) unless stated otherwise.

	Weekday (n = 1644)		Weekend (n = 1642)	
Age, Years (Mean (SD))	13.8	(.5)	13.8	(.5)
Sex (Female)	891	(54.2)	906	(55.2)
Ethnicity (White British)	1310	(79.7)	1312	(79.9)
Eligibility for Free School Meals (Yes)	117	(7.1)	113	(6.9)
Highest Educational Qualification for Mother (NVQ4)	632	(38.4)	629	(38.3)
Attainment 8 (Mean (SD))	56.3	(16.5)	56.9	(16.4)

Note. NVQ, National Vocational Qualification; SD, Standard Deviation.

### Time-use compositions

Table 2 presents the time spent in each behaviour set. On weekdays, participants spent approximately 62% of their time sleeping, 2% in physical activity, 10% using media, 3% spent in school related activities, 4% participation in hobbies, and 18% in domestic activities. On weekends, participants spent approximately 59% of their time sleeping, 14% using media, 8% participating in hobbies, and 19% in domestic activities. After adjustment for covariates, the ILR coordinates for time-use composition were significantly associated with Attainment 8 in both the weekday (p < .001) and weekend (p < .001) samples.

**Table 2:** Descriptive characteristics of time-use compositions (min/day).

	Raw Activity Means (SD)				Geometric Means*	
	Weekday		Weekend		Weekday	Weekend
Sleep	562	(125.21)	616	(125.27)	897	852
Physical Activity	76	(94.13)	72	(104.86)	23	NA
Media	201	(162.93)	275	(191.33)	143	202
School	261	(204.81)	33	(78.13)	50	NA
Hobbies	144	(152.49)	213	(175.83)	61	114
Domestic	197	(124.83)	232	(141.48)	266	272

Note: *Geometric mean adjusted to sum to 1440 min/day. Weekday analysis, n = 1644. Weekend analysis, n = 1642. SD, Standard Deviation.

### Predicted differences in Attainment 8

Table 3 presents the weekday compositional isotemporal substitution analysis, using 20-minute reallocations between behaviour sets. Models that simulated the addition or removal of time from sleep were significantly associated with Attainment 8, with increased sleep duration associated with a lower Attainment 8 score. The simulated addition or removal of time from physical activity was also associated with Attainment 8 scores, wherein the addition of physical activity was associated with higher Attainment 8. The only exception to this trend was the substitution of time from hobbies into physical activity, wherein the lower bound of the 95%CI overlapped zero. The strongest associations were observed in models that simulated the removal of time from physical activity. The substitution of 20 minutes to sleep from physical activity produced the strongest association of −.81, equating to a 1.5% reduction in Attainment 8 score. Simulated increases in media use, school-related activities, or domestic tasks from hobbies were associated with lower Attainment 8 score.

**Table 3:** Predicted difference in Attainment 8 score for reallocations of 20 minutes between behaviour sets (weekday analysis).

Time Reallocation		Attainment 8	
Add 20 Minutes	Remove 20 Minutes	Beta	95% CI*
Sleep	Physical Activity	**−.81**	**(−.87 to −.76)**
Sleep	Media	**−.20**	**(−.25 to −.15)**
Sleep	School	**−.21**	**(−.26 to −.16)**
Sleep	Hobbies	**−.31**	**(−.36 to −.26)**
Sleep	Domestic	**−.18**	**(−.23 to −.13)**
Physical Activity	Sleep	**.29**	**(.24 to .34)**
Physical Activity	Media	**.09**	**(.04 to .14)**
Physical Activity	School	**.09**	**(.03 to .14)**
Physical Activity	Hobbies	−.02	(−.07 to .03)
Physical Activity	Domestic	**.11**	**(.06 to .17)**
Media	Sleep	**.19**	**(.13 to .24)**
Media	Physical Activity	**−.63**	**(−.68 to −.58)**
Media	School	−.02	(−.07 to .03)
Media	Hobbies	**−.13**	**(−.18 to −.07)**
Media	Domestic	.01	(−.05 to .06)
School	Sleep	**.16**	**(.11 to .21)**
School	Physical Activity	**−.65**	**(−.70 to −.60)**
School	Media	−.04	(−.09 to .01)
School	Hobbies	**−.15**	**(−.20 to −.10)**
School	Domestic	−.02	(−.07 to .04)
Hobbies	Sleep	**.24**	**(.19 to .29)**
Hobbies	Physical Activity	**−.** **57**	**(−.** **62 to −.52** **)**
Hobbies	Media	.04	(−.01 to .09)
Hobbies	School	.03	(−.02 to .09)
Hobbies	Domestic	**.06**	**(.01 to** **.11)**
Domestic	Sleep	**.17**	**(.12 to** **.22)**
Domestic	Physical Activity	**−.64**	**(−.70 to −.59)**
Domestic	Media	−.03	(−.08 to .02)
Domestic	School	−.04	(−.09 to .01)
Domestic	Hobbies	**−.14**	**(−.19 to −.09)**

Note: *Significant at p < .05 in bold, 95%CI, 95% confidence interval. Weekday analysis, n = 1644.

Table 4 presents the weekend day compositional isotemporal substitution analysis, using 30-minute reallocations between behaviour sets. The simulated addition or removal of time from sleep was significantly associated with Attainment 8 scores, with increased sleep duration associated with lower Attainment 8. Models that simulated the addition of time to media use were consistently associated with Attainment 8, but with differing directions of association. For example, the reallocation of time to media use from sleep was associated with higher Attainment 8, whereas reallocations from hobbies or domestic tasks into media use were associated with lower Attainment 8. Simulated reallocations of time in sleep, media use, or domestic tasks from hobbies were associated with lower Attainment 8 scores. Overall, the direction and magnitude of associations between behaviour sets and Attainment 8 were comparable between weekday and weekend analyses.

**Table 4:** Predicted difference in Attainment 8 score for reallocations of 30 minutes between behaviour sets (weekend analysis).

Time Reallocations		Attainment 8	
Add 30 Minutes	Remove 30 Minutes	Beta	95% CI*
Sleep	Media	**−.18**	**(−.23 to −.13)**
Sleep	Hobbies	**−.28**	**(−.33 to −.23)**
Sleep	Domestic	**−.24**	**(−.29 to −.19)**
Media	Sleep	**.17**	**(.12 to** **.22)**
Media	Hobbies	**−.12**	**(−.17 to −.07)**
Media	Domestic	**−.07**	**(−.12 to −.02)**
Hobbies	Sleep	**.24**	**(.19 to** **.29)**
Hobbies	Media	**.06**	**(.01 to** **.11)**
Hobbies	Domestic	.00	(−.05 to .05)
Domestic	Sleep	**.22**	**(.17 to** **.27)**
Domestic	Media	.04	(−.01 to .09)
Domestic	Hobbies	**−.06**	**(−.11 to −.01)**

Note: *Significant at p < .05 in bold, 95%CI, 95% confidence interval. Weekend analysis, n = 1642.

### Predicted differences in Attainment 8 with selected time reallocations

**Fig 1:**
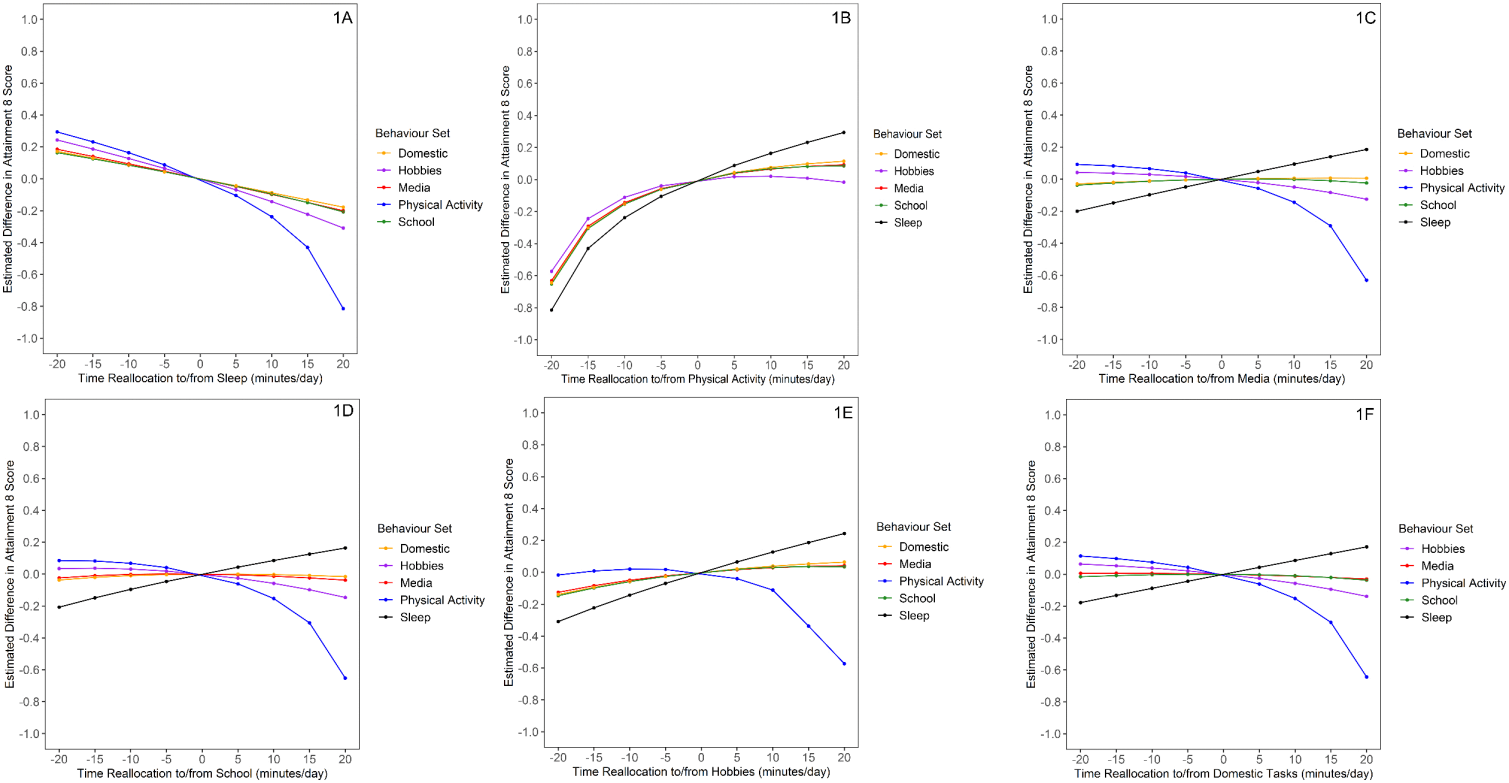
Estimated Differences in Attainment 8 for Weekday Behaviour Reallocations (n = 1644). (1A) Estimated Differences in Attainment 8 for Sleep Reallocations. (1B) Estimated Differences in Attainment 8 for Physical Activity Reallocations. (1C) Estimated Differences in Attainment 8 for Media Reallocations. (1D) Estimated Differences in Attainment 8 for School Reallocations. (1E) Estimated Differences in Attainment 8 for Hobbies Reallocations. (1F) Estimated Differences in Attainment 8 for Domestic Reallocations.

**Fig 2:**
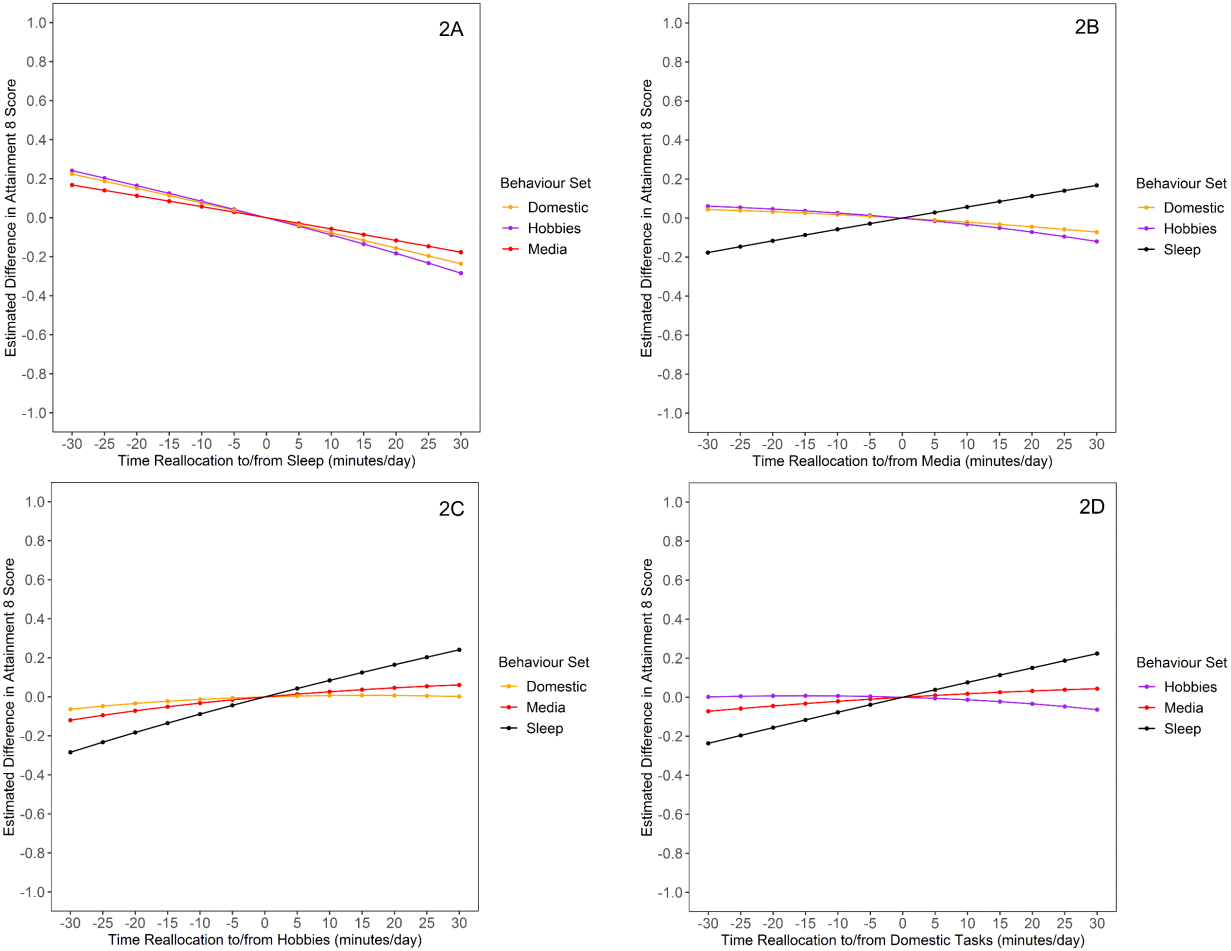
Estimated Differences in Attainment 8 for Weekend Behaviour Reallocations (n = 1642). (2A) Estimated Differences in Attainment 8 for Sleep Reallocations. (2B) Estimated Differences in Attainment 8 for Media Reallocations. (2C) Estimated Differences in Attainment 8 for Hobbies Reallocations. (2D) Estimated Differences in Attainment 8 for Domestic Reallocations.

Predicted differences in Attainment 8 associated with behavioural reallocations in the range of ± 20 minutes (weekday data) and ± 30 minutes (weekend data) are presented in Figs 1 and 2. In both the weekday and weekend analyses, simulated reallocations into sleep from any behaviour set were associated with reduced Attainment 8 in an approximately linear manner (Figs 1A, 2A). In the weekday analysis, there was evidence of a curvilinear (non-symmetrical) association with Attainment 8 for reallocations of time spent in physical activity, wherein modelled reductions in physical activity produced larger predicted differences in Attainment 8 than a modelled increase of the same duration (Fig 1B). Modelled additions and reductions in duration of hobbies, media use, school-related activities, and domestic tasks were linearly associated with Attainment 8, but non-significant in most cases (Figs 1C, 1D, 1E, 1F). In the weekend analysis, modelled additions and reductions in duration of media use, hobbies, and domestic tasks were linearly associated with Attainment 8 (Figs 2B, 2C, 2D).

## Discussion

The present study examined the longitudinal association of 24-hour time-use behaviour with academic attainment. The reallocation of time to sleep from any other behavioural set was associated with lower academic attainment, while comparable increases in physical activity or hobbies were associated with higher academic attainment. Simulated increases in school-related activities or media-use were mostly associated with reduced academic attainment. The strongest associations were observed in models that simulated the removal of time from physical activity in the weekday data. Associations were largely consistent between week and weekend days and remained small in magnitude across all behaviour sets.

A simulated reallocation of 20 minutes into sleep from any other behavioural set was associated with reduced academic attainment in both the weekday and weekend analyses. Our findings differ from a recent cross-sectional analysis in Chinese adolescents, which showed that substituting 50 minutes from video gaming to sleep was associated with enhanced academic attainment [27]. This association, however, was confined only to those participants defined as ‘sleep-restricted’ (<8 hours of sleep per night). In the present study, the majority of participants exceeded 9 hours of sleep per day, which may, in part, account for differing findings. More broadly, our findings are in line with the suggestion that sleep duration outside of an optimum range could be associated with lower academic attainment. For example, adolescents aged 16−19 years who slept between 7 and 9 hours/night achieved a higher grade point average compared to those with either longer (≥9 hours) or short sleep durations (<5 hours [12]). That both insufficient and excessive sleep may negatively impact academic attainment indicates a possible inverted U-shape dose-response association [42], similar to that observed with health-related outcomes [43,44]. An alternative explanation is that longer sleep duration could, in some cases, reflect disengagement or other underlying difficulties among young people at risk of lower academic attainment. However, it should be acknowledged that the 4am-4am assessment protocol deployed for time-use diaries in this study hinders accurate measurement of overnight sleep duration [45]. Future research on this topic should seek to better capture sleep duration, and other sleep components, such as quality and consistency, through alternative self-report protocols or device-based measurement.

In the weekday analysis, the addition of 20 minutes of physical activity was consistently associated with enhanced academic attainment, whilst comparable reallocations of physical activity to any other behaviour set were associated with the largest observed reductions in academic attainment. Our findings are consistent with cross-sectional data from a sample of 1685 Australian children aged 11–12 years, which showed that reallocating time from screen-use to physical activity was associated with higher NAPLAN test scores [26]. The associations of physical activity with academic attainment remained consistent in direction and magnitude regardless of which behaviour set was reallocated, suggesting that the benefits of physical activity for academic attainment may be universal and not dependent on the specific substitution of one or more alternative behaviours. Indeed, the five strongest associations were observed for reallocations that entailed a reduction in time spent in physical activity. This is important, as recent longitudinal research shows device-measured physical activity declines between the ages 9–15 years, with a smaller but continued decline through to 24 years [46]. Given the importance of sustained physical activity for academic attainment and other health-related outcomes, identifying and implementing effective interventions to support continued engagement in physical activity during adolescence is warranted. Emerging evidence suggests that associations between physical activity and academic attainment may differ by activity type, such that individual sports (e.g., swimming) may be more strongly associated with academic attainment than team sports (e.g., football) [47,48]. Relative to team sports, individual sports may more strongly promote psychological skills, such as self-motivation and goal setting, and require a higher level of preparation for training and skill development [49], which may confer benefits in the academic sphere. Future research that examines how substituting time between different physical activity modalities is associated with academic attainment may be beneficial.

Modelled differences in Attainment 8 scores were larger in magnitude for reallocations that reduced physical activity compared to those that increased activity. This asymmetry has also been observed in compositional isotemporal substitution modelling of time-use and its association with mental health outcomes [24] and may suggest that the academic benefits of physical activity plateau after a moderate time increase. This is partially reflected in Fig 1B, where the non-linear association for the potential benefits for academic attainment appear to plateau for reallocations of five minutes or greater. In the present study, the mean weekday physical activity level was approximately 72 minutes, slightly above the recommended 60 minute per day for this population [50]. This finding may suggest that once the recommended level of physical activity has been met, additional activity yields limited benefits to academic attainment, but reducing physical activity to below the recommended level appears to have a more substantial detrimental effect on academic attainment.

Simulated increases in media consumption, which encompassed a range of activities including internet browsing (social media and non-social media sites), watching television, and playing video games, showed a mixed pattern of associations with Attainment 8. For instance, in the weekday data, reallocations to media activities from physical activity, school-related activities, or hobbies were associated with a reduction in academic attainment, whereas substituting time from sleep and domestic tasks was associated with enhanced academic attainment. Previous research has indicated that the association between media use and academic attainment may vary for different screen or media-related behaviours. For example, watching television and playing video games have been associated with lower academic attainment [51,52], while internet use for educational purposes has shown positive associations with attainment [53,54]. Taken together, these findings suggest that composite markers of media or screen behaviour may not be especially insightful for understanding associations with academic attainment and that a focus on specific behaviour types may be more appropriate. This disaggregated approach, however, presents a challenge with regard to obtaining robust data across a breadth of different media-related activities and an analytical challenge wherein isotemporal substitution analyses are limited when individual behaviours are undertaken for short durations.

In the weekday analysis, simulated increases in school-related activities, which included studying in the classroom and completing homework were consistently associated with reduced academic attainment. While our findings may seem counterintuitive, previous research has shown that the relationship between student learning or homework time and academic attainment is non-linear [55,56]. For example, average reading scores on the Programme for International Student Assessment (PISA) were highest among students who reported 24–27 hours of weekly learning time in regular lessons, but declined when the time exceeded 27 hours per week [57]. It may suggest that additional learning time reaches a point of diminishing returns, but it is important to note that these findings may not be uniform across all students. For instance, Liu et al. [55] found that for students in the top 50% of mathematics, science and reading scores, additional learning time yielded minimal benefits. However, for students with lower academic attainment in mathematics, increasing study time was positively associated with attainment. Further research into the potential moderating role of academic ability on the association between time-use and overall academic attainment may be valuable.

Simulated increases in time spent on hobbies were mostly associated with enhanced academic attainment, whilst comparable reductions in time spent on hobbies were consistently associated with reduced Attainment 8. The hobby category encompassed a range of activities, including arts and crafts [58], reading for pleasure [59,60], and musical activities [61], which have been associated with enhanced academic attainment in previous research. For example, reading for pleasure was positively associated with GCSE grades, independent of homework, in a UK sample of 845 adolescents [59]. Reading for pleasure [60] and musical activities [62] have been associated with improved cognitive performance, in the form of memory of picture sequences or sustained attention, respectively, suggestive of a potential mechanism through which these activities may enhance academic attainment. However, it should also be noted that young people aged 10–15 years old from the lowest income backgrounds are almost three times less likely to participate in hobby activities compared to young people from wealthier backgrounds [63]. For example, 32% of young people from the highest-income households take part in musical activities compared to 11% of young people from the lowest-income households. Although we adjusted for markers of socioeconomic position in the present study, it is possible that measurement error in the socioeconomic position markers led to residual confounding. Nevertheless, the present study highlights that providing time for young people to engage in specific types of hobbies may provide opportunities for experiential learning that could further enhance academic attainment.

### Strengths and limitations

Strengths of this study included the large, geographically and demographically diverse sample, with data from 24-hour time-use diaries and academic attainment assessed via formal school examinations. Our study adds to the limited number of longitudinal studies that have used isotemporal substitution analysis to examine associations between time-use behaviours and academic attainment. We acknowledge the following limitations. Data on academic attainment was only available in England; therefore, outcome data could not be obtained from the other UK nations (Scotland, Wales, and Northern Ireland) that were part of the MCS, reducing the available sample for analysis. Time-use diaries are susceptible to recall and social desirability bias, under-reporting of short duration activities, and potential misclassifications due to diary sensitivity [64]. The behaviour sets used in this analysis were composite variables, use of which may have masked unique associations for individual behaviours, such as daytime/overnight sleep or specific physical activity types. However, this is common practice in analyses using time-use data; necessary to manage the volume and interpretability of results and, specifically, ensures exposures of sufficient duration to allow for conduct of isotemporal substitution analyses. Our analysis was based on one randomly selected diary day, which may not reflect typical behaviour patterns; however, restricting assessment to short periods is consistent with established time-use diary methodology designed to minimise participant burden [65]. Behavioural data was limited to a single point of assessment (age 14 years); multiple assessments would have allowed for exploration of associations over differing timeframes and capture of changes in behaviour in the period leading up to assessment of academic attainment. Finally, we acknowledge potential for selection bias in our analytical sample; participants excluded from the analysis, due to missing or invalid diary data, were less likely to be of White ethnicity and more likely to be in receipt of free school meals.

## Conclusion

This study contributes to the growing body of evidence on the associations between adolescents’ time-use and their academic attainment. We found that reallocations to sleep from any other behavioural set was adversely associated with academic attainment, while comparable increases in physical activity or hobbies were beneficially associated with academic attainment. The findings suggest that adolescent time-use at age 14 may be beneficially associated with academic attainment at age 16, but the associations are behaviour-specific and small in magnitude. Future research should continue to explore the associations between time-use and academic attainment to aid intervention development.

## Supporting information

S1 Table Recoding of time-use diary entries into behavioural sets. (PDF)

S2 Table Predicted difference in Number of Passes for reallocations of 20 minutes between behaviour sets (weekday analysis). (PDF)

S3 Table Predicted difference in Number of Passes for reallocations of 30 minutes between behaviour sets (weekend analysis). (PDF)

S1 Fig Participant flow chart. (PDF)
