# Supplementary material for: Adolescents’ time-use and academic attainment: A longitudinal, compositional analysis in the Millennium Cohort Study
Source: PLoS One. 2026 Apr 9;21(4):e0346302. doi: 10.1371/journal.pone.0346302 (PMC13065070; doi:10.1371/journal.pone.0346302)
Supplement: S1 Table — (PDF) [file pone.0346302.s001.pdf]

S1 Table. Recoding of time-use diary entries into behavioural sets.

Behaviour set	Time-use diary: recorded activity
Sleep	Sleeping and resting (including sick in bed)
Physical Activity	Cycling: Individual ball games and training (e.g. tennis, badminton); Jogging, running, walking, hiking; Team ball games and training (e.g. football, hockey); Swimming and other water sports; Other exercise and sports, dancing, keeping fit, skiing, gymnastics; Travel by physically active means (walk, bike, etc.)
Electronic Media	Speaking on the phone (including Skype, video calls); Answering emails, instant messaging, texting; Browsing and updating social networking sites (e.g. Twitter, Facebook, BBM, Snapchat); General internet browsing, programming (not time on social networking sites); Listening to music, radio, iPod, other audio content, Playing electronic video games and Apps; Watch TV, DVDs, downloaded videos
School-related activities	Homework; In class; School breaks; School clubs; Detention
Hobbies and Socialising	Attending live sporting events; Cinema, theatre, performance, gig etc.; Exhibition, museum, library, other cultural events; Shopping (including window shopping, hanging out at a shopping centre); Speaking, socialising face-to-face; Volunteering; Religious activities (including going to places of worship, praying etc.); Did nothing, just relaxing, bored, waiting; Hobbies, arts and crafts, musical activities, writing stories, poetry etc.; Reading (not for school); Other activities not listed
Domestic, personal care, and work-related activities	Personal care (including taking a shower/bath, grooming, getting dressed etc.); Paid work (including paid babysitting and paid work for the family); Unpaid work for family or other non- household members (e.g. help in family business); Cooking, cleaning, and shopping for the household; Fixing things around the house, fixing bike, gardening; Looking after brothers, sisters, other children in the household; Looking after parent or other adult in the households (medical or personal care); Pet care; Eating or drinking in a restaurant or café; Eating a meal; Eating a snack or having a drink; Travel by bus, taxi, tube, plane; Travel by car, van (including vehicles owned by friends and family)
