# Supplementary material for: Adolescents’ time-use and academic attainment: A longitudinal, compositional analysis in the Millennium Cohort Study
Source: PLoS One. 2026 Apr 9;21(4):e0346302. doi: 10.1371/journal.pone.0346302 (PMC13065070; doi:10.1371/journal.pone.0346302)
Supplement: S2 Table — (PDF) [file pone.0346302.s002.pdf]

S2 Table. Predicted difference in Number of Passes for reallocations of 20 minutes between behaviour sets (weekday analysis).

Time Reallocation		Attainment 8	
Add 20 Minutes	Remove 20 Minutes	Beta	95% CI*
Sleep	Physical Activity	**-.115**	**(-.124 to -.102)**
Sleep	Media	**-.030**	**(-.038 to -.020)**
Sleep	School	**-.023**	**(-.031 to -.013)**
Sleep	Hobbies	**-.033**	**(-.042 to -.024)**
Sleep	Domestic	**-.031**	**(-.040 to -.022)**
Physical Activity	Sleep	**.042**	**(.033 to .051)**
Physical Activity	Media	**.013**	**(.004 to .022)**
Physical Activity	School	**.019**	**(.010 to .028)**
Physical Activity	Hobbies	**.009**	**(.000 to .018)**
Physical Activity	Domestic	**.010**	**(.001 to .020)**
Media	Sleep	**.027**	**(.018 to .036)**
Media	Physical Activity	**-.088**	**(-.097 to -.079)**
Media	School	.004	(-.005 to .013)
Media	Hobbies	-.006	(-.015 to .003)
Media	Domestic	.004	(-.013 to .005)
School	Sleep	**.019**	**(.010 to .028)**
School	Physical Activity	**-.096**	**(-.105 to -.087)**
School	Media	**-.010**	**(-.019 to .001)**
School	Hobbies	**-.014**	**(-.023 to -.005)**
School	Domestic	**-.013**	**(-.021 to .003)**
Hobbies	Sleep	**.027**	**(.018 to .036)**
Hobbies	Physical Activity	**-.088**	**(-.097 to -.079)**
Hobbies	Media	-.002	(-.011 to .007)
Hobbies	School	.004	(-.005 to .013)
Hobbies	Domestic	-.004	(-.013 to .005)
Domestic	Sleep	**.030**	**(.021 to .039)**
Domestic	Physical Activity	**-.085**	**(-.094 to -.076)**
Domestic	Media	-.001	(-.008 to .010)
Domestic	School	.007	(-.002 to .016)
Domestic	Hobbies	-.003	(-.012 to .005)

Note: *Significant at p<.05 in bold, 95%CI, 95% confidence interval. Weekday analysis, n=1644.
