# Supplementary material for: Adolescents’ time-use and academic attainment: A longitudinal, compositional analysis in the Millennium Cohort Study
Source: PLoS One. 2026 Apr 9;21(4):e0346302. doi: 10.1371/journal.pone.0346302 (PMC13065070; doi:10.1371/journal.pone.0346302)
Supplement: S3 Table — (PDF) [file pone.0346302.s003.pdf]

S3 Table. Predicted difference in Number of Passes for reallocations of 30 minutes between behaviour sets (weekend analysis).

**Time Reallocations**		**Attainment 8**	
Add 30 Minutes	Remove 30 Minutes	Beta	95% CI*
Sleep	Media	**-.027**	**(-.036 to -.018)**
Sleep	Hobbies	**-.026**	**(-.035 to -.017)**
Sleep	Domestic	**-.045**	**(-.054 to -.036)**
Media	Sleep	**.026**	**(.017 to .034)**
Media	Hobbies	-.001	(-.009 to .007)
Media	Domestic	**-.020**	**(-.029 to -.011)**
Hobbies	Sleep	**.024**	**(.015 to .033)**
Hobbies	Media	-.004	(-.012 to .005)
Hobbies	Domestic	**-.022**	**(-.030 to -.013)**
Domestic	Sleep	**.043**	**(.033 to .051)**
Domestic	Media	**.015**	**(.006 to .024)**
Domestic	Hobbies	**.016**	**(.007 to .025)**

Note: *Significant at p<.05 in bold, 95%CI, 95% confidence interval. Weekend analysis, n=1642.
