# Supplementary figures and images for: Adolescents’ time-use and academic attainment: A longitudinal, compositional analysis in the Millennium Cohort Study
Source: PLoS One. 2026 Apr 9;21(4):e0346302. doi: 10.1371/journal.pone.0346302 (PMC13065070; doi:10.1371/journal.pone.0346302)

**S1 Fig. Participant Flow Chart.**

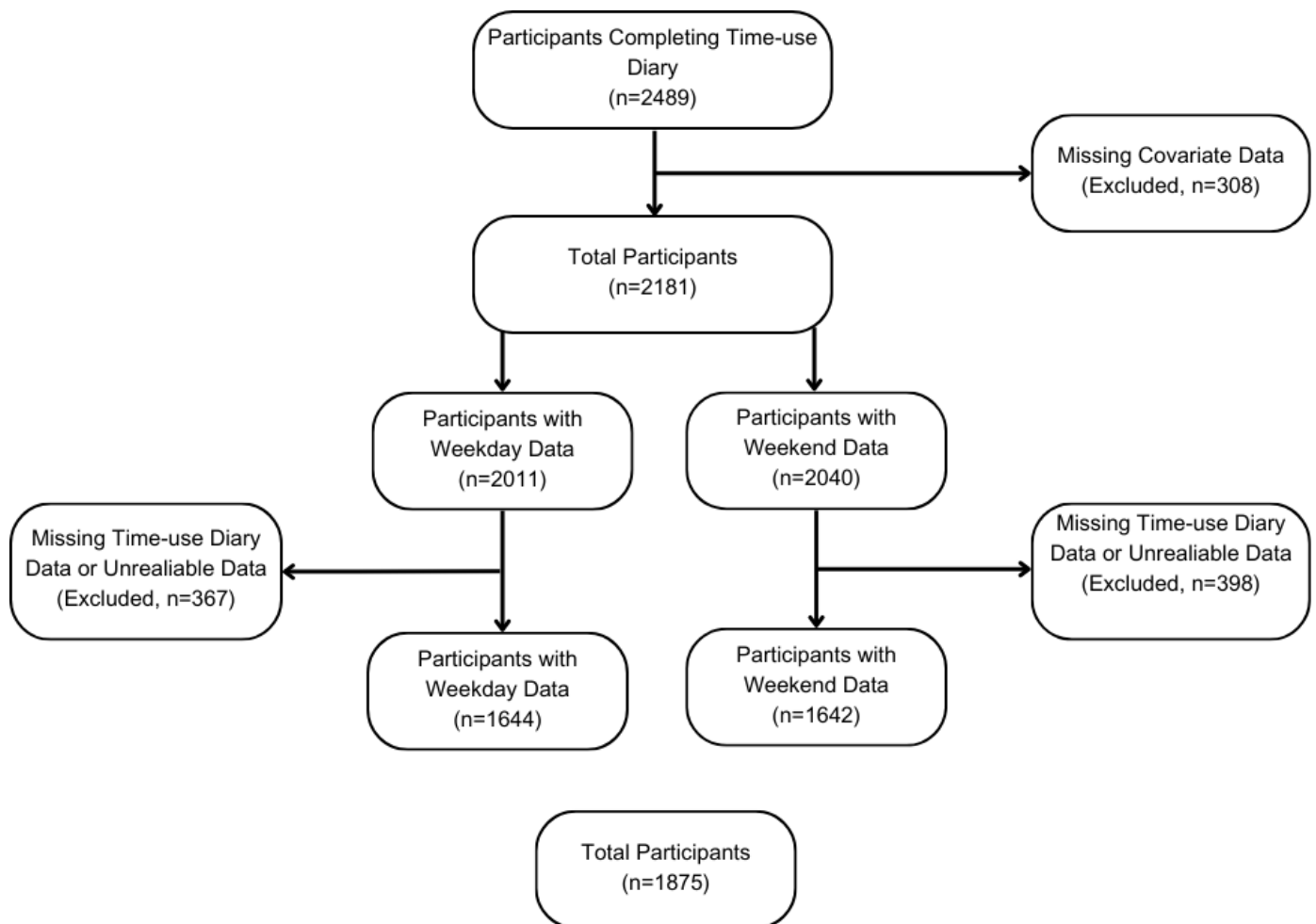

Supplement: S1 Fig — (PDF) [file pone.0346302.s004.pdf]
